# Comparison of mental health screening tools for detecting antenatal depression and anxiety disorders in South African women

**DOI:** 10.1371/journal.pone.0193697

**Published:** 2018-04-18

**Authors:** Thandi van Heyningen, Simone Honikman, Mark Tomlinson, Sally Field, Landon Myer

**Affiliations:** 1 Perinatal Mental Health Project, Alan J. Flisher Centre for Public Mental Health, Department of Psychiatry and Mental Health, University of Cape Town, Cape Town, South Africa; 2 Division of Epidemiology & Biostatistics, School of Public Health and Family Medicine, University of Cape Town, Cape Town, South Africa; 3 Department of Psychology, Stellenbosch University, Stellenbosch, South Africa; 4 Lead investigator of the Centre of Excellence in Human Development, University Witwatersrand, Johannesburg, South Africa; Western Sydney University, AUSTRALIA

## Abstract

**Background:**

Antenatal depression and anxiety disorders are highly prevalent in low and middle-income countries. Screening of pregnant women in primary care antenatal settings provides an opportunity for entry to care, but data are needed on the performance of different screening tools. We compared five widely-used questionnaires in a sample of pregnant women in urban South Africa.

**Method:**

Pregnant women attending a primary care antenatal clinic were administered five tools by trained research assistants: the Edinburgh Postnatal Depression Scale (EPDS), the Patient Health Questionnaire (PHQ-9), the Kessler Psychological Distress scale (K10) and a shortened 6-item version (K6), the Whooley questions and the two-item Generalised Anxiety Disorder scale (GAD-2). Following this, a registered mental health counsellor administered the MINI Plus, a structured clinical diagnostic interview. The Area Under the Curve (AUC) from Receiver Operator Characteristic curve analysis was used to summarise screening test performance and Cronbach’s α used to assess internal consistency.

**Results:**

Of 376 participants, 32% were diagnosed with either MDE and/or anxiety disorders. All five questionnaires demonstrated moderate to high performance (AUC = 0.78–0.85). The EPDS was the best performing instrument for detecting MDE and the K10 and K6 for anxiety disorder. For MDE and/or anxiety disorders, the EPDS had the highest AUC (0.83). Of the short instruments, the K10 (AUC = 0.85) and the K6 (AUC = 0.85) performed the best, with the K6 showing good balance between sensitivity (74%) and specificity (85%) and a good positive predictive value (70%). The Whooley questions (AUC = 0.81) were the best performing ultra-short instrument. Internal consistency ranged from good to acceptable (α = 0.89–0.71). However, the PPV of the questionnaires compared with the diagnostic interview, ranged from 54% to 71% at the optimal cut-off scores.

**Conclusions:**

Universal screening for case identification of antenatal depression and anxiety disorders in low-resource settings can be conducted with a number of commonly used screening instruments. Short and ultra-short screening instruments such as the K6 and the Whooley questions may be feasible and acceptable for use in these settings.

## Introduction

Common perinatal mental disorders (CPMD) are highly prevalent and contribute significantly to maternal morbidity and mortality [1–3]. Although estimates vary between regions, the prevalence rates reported in low and middle income countries (LMIC) are consistently higher than in high income countries (HIC) [3]. There is a high comorbidity between perinatal depression and anxiety disorders, with research showing antenatal anxiety disorders are as prevalent as antenatal depression [4]. CPMD are disabling for maternal functioning and have well-documented, negative consequences for child health and development [5,6].

Despite this, CPMD in LMIC often remain undetected and untreated, leaving a treatment gap of up to 80% [7]. This is partly due to the low levels of recognition by health care workers, especially in poorly-resourced primary-care settings facing high patient volumes [8]. In such contexts, routine, primary care tasks are increasingly being shifted to non-specialist health workers who may have limited training and skills. To address this, the World Health Organisation (WHO) suggests the integration of mental health services into primary care, with increasing advocacy for universal, routine screening at primary care level [9].

Although guidelines have been established in HIC regarding mental health screening for pregnant and post-partum women for depression [10–12], there are no evidence-based guidelines for LMICs. Evidence-based approaches are needed for early identification, treatment and prevention of the negative consequences of CPMD. However, there are concerns about advocating the routine use of screening questionnaires to detect mental disorders in low-resource primary care settings [13,14]. The main argument against routine screening cites the extra burden placed on resource-poor, overburdened health care systems by incorrectly identifying cases (i.e. false negatives) [15], and the ethical considerations of screening where there is no follow-up mental health care [16]. Referral and care pathways for mothers experiencing CPMDs are scant in LMICs, including South Africa, and where mental health services are available, these tend to be restricted and focussed on those with severe mental illness, such as psychosis [17]. If and when CPMD’s are detected, South African women accessing public health services may be referred to a psychiatric nurse, but if this services is available, it is usually accessed at a separate site and not integrated with antenatal services [18].

For many women in LMICs, however, antenatal care provision representes a period where there are relatively high levels of contact between vulnerable mothers and the health system. This is particularly true in South Africa which has a national rate for any antenatal contact of 90.7% [19]. Screening at this point can provide a crucial opportunity for contact with mental health services or other appropriate care options available in the area [3,18,20,21]. A stepped-care approach, where initial screening is followed by more in-depth screening or assessment may mitigate the burden of false positives by reducing the number of incorrectly diagnosed individuals being referred for treatment [18,22].

A number of mental health screening questionnaires have been created over the last two decades, but notably, these have been designed and developed in HICs. Research has shown that short and ultra-short screening instruments, defined as having between 5 and 14 items, and <5 items respectively are, generally, as accurate as the longer instruments [23,24]. These instruments may have an advantage over longer ones since they can be administered in a much shorter time, which is of practical benefit for busy, primary care antenatal environments where health workers may be overwhelmed with high patient volumes and numerous tasks to perform.

There are concerns, however, about the application of screening tools developed in HICs to LMIC populations and contexts [13]. There is a scarcity of cross-culturally validated mental health screening tools, especially for use during pregnancy. There is a paucity of data examining the value of these questionnaires in low-resource, primary care settings in LMICs, and few screening tools have been validated for use in African countries [13].

It is clear that more research is required to develop, refine and rigorously assess the predictive validity and reliability of tools used for assessment of CPMD in LMIC, and to gauge instruments’ performance in detecting both depression and anxiety as well as psychological distress [25]. Most tools have been developed to detect depression, but anxiety disorders are also highly prevalent, with significant negative impact on maternal health and the health and development of offspring. To address these gaps, we compared the psychometric performance of commonly used screening tools to detect Major Depressive Episode (MDE) and/or anxiety disorders in a sample of low-income women in a resource-poor, primary-level antenatal setting.

## Materials and methods

We conducted a cross-sectional study, within the context of a larger study investigating the development of a screening instrument for CPMD, for use in low resource, primary level antenatal settings by non-specialist health workers. Details of the study methodology have been previously described [26].

### Setting

The study was located at a primary care antenatal clinic within a busy urban Community Health Centre (CHC) in the Cape Town metropole. The clinic serves an area with a population of roughly 35 000 people where the majority face high levels of socioeconomic adversity.

### Sample

Every third woman attending their first antenatal visit was invited to participate in the study through systematic sampling. Those who were aged below 18 years or unable to converse in any of the three local languages (English, Afrikaans or isiXhosa) were excluded.

### Data collection

All subjects who provided written consent to participate were screened in two stages. The first stage comprised a brief sociodemographic questionnaire, followed by verbal administration of five, commonly-used mental health screening questionnaires in a face-to-face interview. This was conducted by a field-worker with an honours degree in Psychology and four years of experience as a research assistant in clinical settings. The second stage comprised a structured diagnostic interview carried out by a registered counsellor, with a Bachelor of Psychology (Honours) degree in counselling. Both were trained and supervised by a clinical psychologist. The field worker administered all the English (n = 312) and isiXhosa (n = 37) screens. The registered counsellor administered the screens in Afrikaans to 27 participants. The field worker provided interpreting during the diagnostic interview for the isiXhosa-speaking participants.

### Screening instruments

We chose five screening questionnaires for evaluation, based on their widespread use in detecting CPMD and their brevity (the maximum length of the questionnaires was 10 questions) namely: the Edinburgh Postnatal Depression Scale (EPDS), the Patient Health Questionnaire (PHQ-9), the Kessler Psychological Distress scale (K10), the two-item Generalised Anxiety Disorder scale (GAD-2) and the Whooley questions. The questionnaires were consistently administered in the order that they appear above. Further details on the screening instruments can be found in Table 1. Questionnaires that were not available or validated in isiXhosa or Afrikaans were professionally translated and back-translated for this study by translators at Stellenbosch University Language Centre.

**Table 1:** Depression and anxiety symptom-screening instruments included in the study.

Screening instrument	Measure	Scoring system	No of items	Validated for use in Pregnancy	Validated in South Africa (Population) [Languages]	Recommended cut-off score*
Edinburgh Postnatal Depression Scale (EPDS)	Depression & anxiety	Likert	10	Yes	Yes (Perinatal) [English, isiXhosa]	Varies between >10 and ≥ 15 depending on the setting and whether antenatal or postnatal population
Kessler (K10)	Depression & anxiety	Likert	10	Yes	Yes (General & perinatal) [English]	> 20
Patient Health Questionnaire (PHQ9)	Depression & anxiety	Likert	9	Yes	Yes (General) English, Afrikaans]	Mild: 5–9 Moderate: 10–14 Mod-severe: 15–20 Severe: >20
Two-item Generalised Anxiety Scale (GAD-2)	Anxiety	Likert	2	Yes	Yes (General) [English]	≥ 3
Whooley questions	Depression	Binary	2 with an optional “help” question	Yes	Yes (Antenatal) [English]	≥ 1 without the help question, or ≥ 1 + “yes” with the help question

* As recommended by literature

The EPDS has been found to be a reliable instrument in screening for antenatal and postnatal depression [13,27,28] and has been validated for use in wide range of settings including South Africa [29–31], and for verbal administration by an interviewer in both English and isiXhosa [30]. A threshold score of ≥11 on the EPDS has been shown to have a sensitivity of 80% and specificity of 76% for major and minor depression combined, in a South African context [31]. Additionally, three EPDS items (items 3, 4, and 5: EPDS-3A) have been found to cluster together to indicate probably anxiety in perinatal women, with an ability to distinguish reliably depression from anxiety in some studies and with an optimum cut-off of 6 or more (possible range: 0–9) [32]. Given this evidence from elsewhere, we decided to include examination of the performance of this subscale for anxiety in our sample. There are a number of studies that have investigated the performance of short and ultra-short versions of the EPDS, ranging from 7-item to 2-item versions [23,24,33].

The PHQ-9 was developed from the Primary Care Evaluation of Mental Disorders Procedure (PRIME-MD) for detection of depression in primary care settings [34,35]. It has been tested for validity among diverse populations, including in South Africa for use by an interviewer [36,37]. It has been validated elsewhere in antenatal and postnatal settings [34,38,39]. Scores are interpreted as follows: 0–4 normal, 5–9 mild depressive symptoms, 10–14 moderate depressive symptoms ≥15 moderately severe to severe depressive symptoms. A two-item version, with a cut-off of ≥3, has shown favourable performance in detecting depression, and is advocated for use as an initial screen in busy clinical settings [40,41].

The K10 [42] has been found to have reasonable sensitivity and specificity in detecting depression, PTSD, panic disorder and social phobia and may be a useful screening measure for mood and anxiety disorders in perinatal women [43,44]. A score of ≥ 21.5 (sensitivity, 0.73; specificity, 0.54) has been determined as the best screening cut-off for Structured Clinical Interview for DSM-V (SCID)-defined depressive and anxiety disorders amongst pregnant women in South Africa [44]. It was validated for use in South Africa, in both English and Afrikaans, and for interviewer administration [43]. A six-item version, the K6, is also advocated to screen for common mental disorders in primary care, and has been validated in perinatal populations in low income settings [8,43,45].

The GAD-2 is a 2-item form of the GAD-7, which asks patients how often over the last two weeks they were ‘bothered by (1) feeling nervous, anxious, or on edge and (2) not being able to stop or control worrying’ [46]. It has not yet been validated for use in South Africa or with antenatal populations, but is regarded as being a clinically useful, ultrashort screening tool for Generalised Anxiety Disorder (GAD) and other anxiety disorders in primary care [46]. The GAD-2 uses a Likert scoring system, with scores ranging from 0 to 6. A score of three or more is considered a “positive” screen, with a sensitivity of 65% and specificity of 88% for any anxiety disorder [46]. Recently updated NICE guidelines advocate the use of the GAD-2 to screen for anxiety in primary care settings, including the perinatal population [11,47]. This tool is administered during an interview [48].

The Whooley questions, are two, binary-scoring questions to screen for depression, which, like the PHQ, were also adapted from the PRIME-MD [48]. These offer a relatively quick and convenient way of case-finding for non-specialist health workers in primary care settings [48]. The United Kingdom’s National Institute for Clinical Excellence (NICE) guidelines recommend that healthcare professionals ask these two mood symptom questions at a woman’s first contact with primary level antenatal or postnatal care [10,49]. An answer of “yes” to either of the two Whooley questions is considered a “positive” screen. An additional “help” question may be asked if the woman responds positively to either of the first two questions [10]. The Whooley questions have recently been validated for use in the South African antenatal population, with an optimal score of ≥ 2 yielding a sensitivity of 73% and specificity of 76% [50].

### Diagnostic data

The Mini-international Neuropsychiatric Interview plus (MINI Plus) diagnostic interview Version 5.0.0 was used to obtain diagnostic data. The MINI Plus is a short, structured, diagnostic interview which is used to diagnose the major axis I psychiatric disorders in DSM-IV and ICD-10 [51]. It has been validated for use in South Africa [52] and validated translations were used for non-English speaking participants in this study [44,53]. The MINI Plus was chosen because it has been used extensively in research in LMIC settings and is relatively short and easy to administer [54].

### Ethics and referrals

The study was approved by the Faculty of Health Sciences Human Research Ethics Committee at the University of Cape Town (HREC REF: 131/2009), and by the Western Cape Department of Health Provincial Research Committee (ref: 19/18/RP88/2009). Participants who received a diagnosis of MDE or anxiety on the MINI Plus or who met the cut-off on EPDS were referred for counselling. Those who were diagnosed with severe psychopathology, or who expressed suicidal ideas or plans, were referred to the emergency psychiatric services at the CHC.

### Data analysis

Data were analysed using Stata version 13.1. Although diagnostic data were collected on several different types of psychopathology, for the purposes of this paper, we selectively analysed the data for current MDE and current anxiety disorders (including post traumatic stress disorder (PTSD), generalised anxiety disorder (GAD), obsessive compulsive disorder (OCD), panic disorder, social anxiety disorder and specific phobia). As we were interested in how the screening tools performed in detecting both antenatal anxiety and antenatal depression, the sample was categorised into cases vs. non-cases based on the result of the MINI Plus, whereby any participant with a MINI diagnosis of MDE or any anxiety disorder was classified as being a “case”.

We also assessed current suicidal ideation and behaviour (SIB) using the MINI Plus module on suicidality. We used the Statistics South Africa definition of the poverty line as a monthly income of R501 (34 US$) or less per month [55].

The internal consistency of each screening instrument was estimated using Cronbach’s α statistic [56]. Pearson’s correlation matrix was used to assess the strength of correlation between screening instruments. Sensitivity and specificity, positive and negative predictive values and likelihood ratios for various cut-off scores for each screening instrument were analysed using receiver operating characteristic (ROC) curves. The overall predictive value of instruments were assessed by looking at the area under the curve (AUC), and optimal cut-off scores were plotted along the curve. The choice of cut-off scores at which MDE diagnosis could be made were determined by balancing sensitivity, specificity, positive predictive values (PPV) and percentage correctly classified in order to minimise the number of false positives. See Table in S1 Table. All analyses were conducted against the three diagnostic categories, namely MDE; anxiety; and MDE and/or anxiety, and were disaggregated into sociodemographic categories for age, education level and language.

## Results

### Participants

Of 617 women approached, 135 declined participation, 58 were ineligible and 376 participants were included in the study. Logistical barriers to participation were the most frequent reasons provided for non-participation. These included not being able to take time off work, not having childcare for the time needed for the interview and needing to leave with pre-arranged transport. The average age was 26.8 years (SD 5.9 years, range 18–48 years). The majority were unmarried but in a stable relationship (51%). More than half of participants (55%) were unemployed, and 43% were living below the poverty line, indicating that they would probably have to sacrifice some food items to be able to afford to buy essential non-food items [55]. Three quarters (75%) were in their second or subsequent pregnancy, with 20% of pregnancies being reported as both unplanned and unwanted. Table 2.

**Table 2:** Description of the sample.

	Total sample N (SD)	MDE diagnosis N (%)	Anxiety diagnosis N (%)	MDE and/or Anxiety diagnosis N (%)
Mean age (SD)*	26.8 (5.9)	26.1 (6.3)	27.4 (5.6)	26.7 (5.9)
Highest level of education ≤ Grade 10	151 (40)	38 (47)	37 (43)	56 (46)
> Grade 10**	225 (60)	43 (53)	49 (57)	66 (54)
Relationship status: Married	146 (39)	23 (28)	36 (42)	45 (37)
Stable partner	192 (51)	46 (57)	43 (50)	64 (53)
Casual partner	16 (4)	4 (5)	2 (2)	4 (3)
No partner**	22 (6)	8 (10)	5 (6)	9 (7)
Ethnicity: Black African	133 (35)	21 (26)	25 (29)	35 (29)
“Coloured”^§^	224 (60)	56 (69)	55 (64)	79 (65)
White & “other”**	18 (5)	4 (5)	6 (7)	8 (6)
Employment type: Unemployed	208 (55)	52 (64)	46 (53)	71 (58)
Informal/hawker	17 (5)	1 (1)	4 (5)	4 (3)
Contract/part-time	51 (14)	17 (21)	14 (16)	21 (17)
Full time**	100 (26)	11 (14)	22 (26)	26 (22)
SES: Below poverty line	162 (43)	42 (52)	35 (41)	55 (45)
Above poverty line**	214 (57)	39 (48)	51 (59)	67 (55)
Housing type: Shack dwelling	60 (16)	11 (14)	13 (15)	18 (15)
Backyard dwelling	85 (23)	23 (28)	27 (31)	36 (30)
Formal house	100 (27)	23 (28)	21 (24)	31 (25)
Council house/flat	121 (32)	22 (27)	22 (26)	33 (27)
Other**	10 (3)	2 (3)	3 (4)	4 (3)
Gravidity: Primigravida	96 (26)	19 (23)	9 (10)	23 (19)
Multigravida**	280 (75)	62 (77)	77 (90)	99 (81)
Parity:Nulliparous	122 (32)	26 (32)	20 (23)	35 (29)
Primiparous	128 (34)	24 (30)	29 (34)	38 (31)
Multiparous**	126 (34)	31 (38)	37 (43)	49 (40)
Gestation1^st^ Trimester	96 (32)	21 (22)	24 (25)	34 (35)
2^nd^ Trimester	175 (58)	33 (19)	35 (20)	49 (28)
3^rd^ Trimester	29 (10)	7 (24)	11 (38)	13 (45)
Unplanned & unwanted pregnancy**	74 (20)	24 (30)	22 (26)	31 (25)
History of mental health problems**	57 (15)	30 (37)	32 (37)	37 (30)
Suicidal ideation and behaviour**	69 (18)	23 (33)	24 (35)	32 (46)
Current use of Alcohol**	50 (13)	16 (20)	17 (20)	22 (18)
Current use of substance other than alcohol**	23 (6)	12 (15)	10 (12)	13 (11)
Mean EPDS score*	10.8 (5.7 SD)	17.4 (4.1 SD)	15.1 (5.6 SD)	15.4(5.2 SD)
Mean K10 score*	10.4 (8.5 SD)	20.5 (7.8 SD)	16.9 (8.8 SD)	17.8 (8.5 SD)
Mean PHQ9 score*	7.4 (5.7 SD)	14.1 (5.4 SD)	11.7 (5.9 SD)	12.3 (5.8 SD)
Mean Whooley score*	0.8 (0.9 SD)	1.8 (0.6 SD)	1.4 (0.8 SD)	1.5 (0.8 SD)
Mean GAD-2 score*	1.3 (1.7 SD)	3.1 (1.9 SD)	2.5 (2.1 SD)	2.5 (2.1 SD)

* Two-sample t-test

** Fisher’s Exact test

^**§**^ The term “Coloured” refers to a heterogeneous group of people of mixed race ancestry that self-identify as a particular ethnic and cultural grouping in South Africa. This term, and others such as “White”, “Black/African” and “Indian/Asian” remain useful in public health research in South Africa, as a way to identify ethnic disparities, and for monitoring improvements in health and socio- economic disparities.

Based on the MINI Plus data, 32% of participants were diagnosed with either MDE and/or anxiety disorders. Among these, anxiety disorders were the most frequent diagnosis (23%) followed by MDE (22%). A total of 45 participants (12%) had a comorbid diagnosis of both MDE and anxiety. There were 69 participants (18%) who indicated current SIB on the MINI Plus.

### Internal consistency of scales

The K10, K6, PHQ9 and Whooley questions (with the help question) showed good internal consistency (Cronbach’s alphas >0.8) while the EPDS, the Whooley questions (without the help question) and the GAD-2 demonstrated slightly lower levels of internal consistency (Cronbach’s alpha <0.8 and >0.7). The EPDS-3, the PHQ2 and the anxiety subscale of the EPDS had relatively poor internal consistency (Cronbach’s α <0.6 and >0.5).

On comparing the performance of the EPDS, K10, PHQ9 and Whooley in detecting MDE and/or anxiety disorders by age, education level and language, the only significant finding was that when conducted in isiXhosa, the EPDS performed poorly (measured by AUC; *p* = 0.021) compared to when conducted in the other two languages. Table 3.

**Table 3:** Comparative performance of four screening instruments in detecting CPMD by age, education level & language in a sample of 376 pregnant women.

	EPDS				K10				PHQ9				Whooley				GAD-2			
N = 376	Mean score	Screening prevalence cut point ≥13 (%)	AUC	*p-*value comparing AUC	Mean score	Screening prevalence cut point >20 (%)	AUC	*p*-value comparing AUC	Mean score	Screening prevalence cut point >5 (%)	AUC	*p*-value comparing AUC	Mean score	Screening prevalence cut point ≥1 (%)	AUC	*p*-value comparing AUC	Mean score	Screening prevalence cut point ≥3 (%)	AUC	*p*-value comparing AUC
Total sample	10.6	147 (39)	0.83		10.5	58 (15)	0.84		7.54	211 (56)	0.83		1.1	193 (51)	0.80		1.3	77 (20)	0.76	
Age 18–24	10.8	61 (42)	0.86		10.8	22 (38)	0.89		7.9	85 (40)	0.89		0.8	77 (40)	0.82		1.3	54 (21)	0.77	
Age 25–29	11.1	46 (31)	0.83		11.1	19 (33)	0.83		7.6	66 (31)	0.79		0.9	66 (34)	0.77		1.2	22 (21)	0.78	
Age ≥30	10.5	40 (27)	0.80	0.541	9.3	17 (29)	0.81	0.227	6.7	60 (29)	0.83	0.176	0.7	50 (26)	0.79	0.713	1.0	1 (10)	0.75	0.866
Education ≤Gr 10	11.6	68 (46)	0.84		11.3	32 (55)	0.86		8.1	87 (41)	0.88		0.8	76 (39)	0.79		1.4	180 (60)	0.77	
Education ≥Gr 10	10.3	79 (54)	0.82	0.766	9.8	26 (45)	0.83	0.598	7.1	124 (59)	0.81	0.094	0.8	117 (61)	0.79	0.981	1.2	45 (58)	0.76	0.893
English	9.9	45 (31)	0.81		9.5	20 (34)	0.84		7.2	80 (38)	0.79		0.8	66 (34)	0.79		1.3	27 (35)	0.76	
Afrikaans	11.7	53 (36)	0.91		12.1	23 (40)	0.85		7.9	69 (33)	0.87		0.8	64 (33)	0.81		1.4	30 (39)	0.78	
isiXhosa	11.0	49 (33)	0.79	0.021*	9.7	15 (26)	0.88	0.813	7.4	62 (29)	0.87	0.263	0.8	63 (33)	0.78	0.926	1.1	20 (26)	0.75	0.862

* Shows significance at *p* < 0.05

Pearson’s correlation coefficient showed moderate to strong correlations between screening instruments, ranging from 0.60 (GAD and EPDS) to 0.73 (K10 and EPDS).

### Case-detecting properties of screening tools

Screening tool performance in detecting cases of MDE, anxiety or both MDE and anxiety is presented in Table 4. Optimal cut-off scores and corresponding sensitivity and specificity are presented along with predictive values, likelihood ratios and the percentage of women who would screen positive using that specific cut-off score.

**Table 4:** Comparison of the performance of screening instruments against MINI defined MDE and anxiety diagnoses.

Screening tool	Mini diagnosis	AUC°	Cut point	Correctly classified	Sensitivity	Specificity	LR+°	LR-	PPV^ω^	NPV^∞^	Screening prevalence (%)
EPDS	MDE	0.91	14	82%	86%	81%	4.6	0.2	56%	96%	125 (33)
3-item EPDS*	MDE	0.85	3	76%	83%	74%	3.1	0.2	46%	94%	145 (39)
K10	MDE	0.89	15	84%	81%	85%	5.5	0.2	60%	94%	110 (29)
K6^§^	MDE	0.88	10	85%	78%	87%	6.0	0.3	62%	93%	101 (27)
PHQ9	MDE	0.89	10	82%	79%	82%	4.5	0.3	55%	93%	116 (31)
PHQ2	MDE	0.84	3	80%	65%	84%	3.9	0.4	51%	90%	103 (27)
Whooley	MDE	0.86	2	84%	83%	85%	5.5	0.2	60%	95%	112 (30)
Whooley + help	MDE	0.88	2	81%	89%	79%	4.2	0.1	53%	96%	135 (36)
											
EPDS-3A **	Anxiety	0.69	5	61%	67%	59%	1.6	0.6	33%	86%	178 (47)
K10	Anxiety	0.77	11	72%	76%	70%	2.5	0.3	43%	91%	151 (40)
K6	Anxiety	0.77	8	75%	69%	76%	2.9	0.4	46%	89%	128 (34)
GAD-2	Anxiety	0.73	2	72%	64%	74%	2.5	0.5	43%	87%	129 (34)
											
EPDS	MDE &/ Anxiety	0.83	13	77%	75%	78%	3.4	0.3	62%	87%	146 (39)
3-item EPDS	MDE &/ Anxiety	0.79	3	75%	70%	77%	3.0	0.4	59%	84%	145 (39)
K10	MDE &/ Anxiety	0.85	11	80%	80%	79%	3.8	0.2	65%	89%	151 (40)
K6	MDE &/ Anxiety	0.85	8	81%	74%	85%	4.9	0.3	70%	87%	128 (34)
PHQ9	MDE &/ Anxiety	0.84	10	80%	66%	76%	4.8	0.4	70%	84%	116 (31)
PHQ2 ^§§^	MDE &/ Anxiety	0.78	2	71%	75%	69%	2.5	0.4	54%	85%	171 (45)
Whooley	MDE &/ Anxiety	0.79	2	80%	66%	87%	5.2	0.4	71%	84%	112 (30)
Whooley + help	MDE &/ Anxiety	0.81	2	79%	73%	82%	4.1	0.3	66%	86%	135 (36)

* The 3-item EPDS is an ultra-brief version of the EPDS, which includes items 2, 9 and 10.

** The EPDS-3A is the anxiety sub-scale of the EPDS and includes items 3, 4 and 5.

^§^ The K6 is a shorter version of the K10 and includes items 2, 4, 5, 8, 9 and 10.

^§§^ The PHQ2 is an ultra-brief version of the PHQ9, which includes items 1 and 2.

° AUC: Area under the receiving operating characteristic (ROC) curve.

^ω^ LR+: Positive likelihood ratio, LR-: negative likelihood ratio.

^∞^ PPV: positive predictive value, NPV: negative predictive value.

### Detecting MDE

For detecting MDE, all screening tools performed well with AUC ranging from 0.84 to 0.91. The EPDS had the highest AUC, followed by the K10 and PHQ9. Of the ultra-short screening tools, the K6 and the Whooley questions with the help question included, had the highest AUC’s, showing that they are highly accurate instruments. In this setting, the EPDS and K6 were the best performing tools to detect MDE.

For detecting depression, the EPDS performed the best. The ultra-short Whooley questions, with AUC of 0.86 and 84% correctly classified, performed comparably to the longer K10 and K6. Their screening prevalence was lower than EPDS and only marginally higher than K10 and K6. Adding the help item to the two Whooley questions improved overall performance marginally to 0.88 (AUC) and increased sensitivity by 6% but decreased PPV, thereby increasing the number of false positives. The poorest performing tools were the 3-item EPDS and the PHQ2. These had the lowest AUC, sensitivity and percentage correctly classified, as well as the poorest likelihood ratios and the highest screening prevalences (45%).

### Detecting anxiety disorders

For detecting anxiety disorders, the K10 and K6 had the highest AUCs at 0.77, followed by the GAD (AUC = 0.73). The K10 had the highest sensitivity, followed by the K6, which had the highest specificity. Both the K10 and GAD-2 had high numbers of false positives (screening prevalence was almost double the actual prevalence of 22%). The K6 appeared to perform best overall. The EPDS anxiety subscale performed worst, with an AUC of 0.69. In this regard, caution should be exercised in using the EPDS to detect anxiety as the instrument was explicitly designed to measure depressive symptomatology.

### Detecting MDE and anxiety disorders

**Fig 1:**
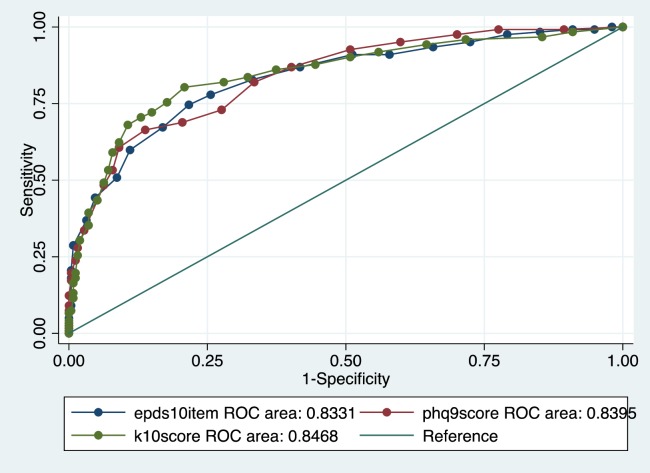
Roc comparison of performance of brief screening tools for detecting MDE and/or anxiety disorders.

**Fig 2:**
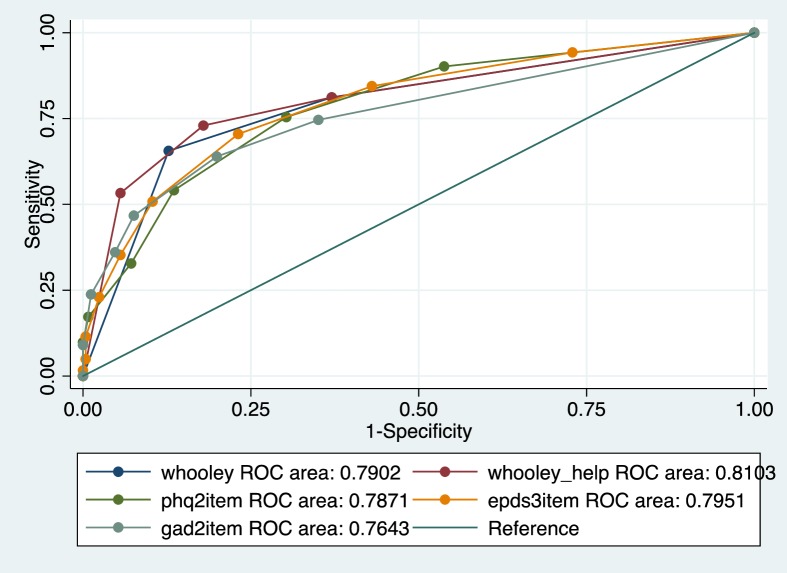
Roc comparison of performance of ultra-brief screening tools for detecting MDE and/or anxiety disorders.

For detecting both MDE and anxiety, the EPDS, K10, K6, PHQ9 and Whooley questions with help question added, had AUC = 0.81 to 0.85, while the two Whooley questions, PHQ2 and 3-item EPDS had AUC = 0.78 and 0.79. The K10 had the highest sensitivity and the Whooley without the help question had the highest specificity. Adding the “help” question enhanced the performance of the Whooley by 0.2 points on the AUC, and increased the sensitivity of the tool. However, it decreased specificity and increased the number of cases screened as positive by 6%, which may add an unnecessary burden of false positives in a clinical setting. (Figs 1 and 2).

## Discussion

The purpose of the study was to compare the performance of five screening tools in detecting CPMD in a sample of low-income, pregnant women at a primary level antenatal facility. We found few material differences in the performance of depression screening tools in identifying pregnant women with MDE. This finding is in line with previous studies, that analysed the performance of such tools in detecting antenatal depression in LMIC [25] and with another study comparing screening tool performance in a non-pregnant population [8]. However, in this setting, the performance of tools in detecting anxiety disorders and in detecting both disorders simultaneously was less optimal.

The EPDS was the best performing instrument for detecting MDE and the K10 and K6 for anxiety disorder. For MDE and/or anxiety disorders, the EPDS had the highest AUC (0.83). Of the short instruments, the K10 (AUC = 0.85) and the K6 (AUC = 0.85) performed the best, with the K6 showing good balance between sensitivity (74%) and specificity (85%) and a good positive predictive value (70%).

In this context, symptoms of anxiety are as prevalent as symptoms of depression among pregnant women [57]. The five commonly used screening tools did not perform well at detecting antenatal anxiety disorders separately from MDE. However, only one out of these five tools, the GAD-2, was specifically designed to measure anxiety. In addition, due to the high prevalence of both MDE and anxiety disorders, it is unlikely that women will be screened for anxiety disorders without also screening for depression. The most feasible and practical tool would be one that performs well in screening for symptoms of both disorders, and identifying effective screening tools for both anxiety and depression remains a challenge for the field.

Criteria used for determining the best performing screening tools included the psychometric properties of the individual tools as well as the practical application of these tools in a busy, low resource setting. We considered the ideal characteristics of a screening tool to be one that; had relatively few items, had good sensitivity, had good predictive value and had a good AUC. The tools that met these criteria and produced screening rates that were close to actual diagnostic prevalence were seen as the most feasible and practical tool for this setting. Although the EPDS had the highest AUC, the K6 showed a good balance between sensitivity and had the highest PPV and percentage correctly classified out of all the tools. Finally, the percentage that would screen positive with the K6 was 27%, which was lower than the EPDS (33%), and closest to the actual diagnostic prevalence of 21%. Out of the short instruments examined, the K10 and K6 appear to be the tools that meet most of these criteria, with the best overall performances across the diagnostic categories. Fig 1.

Out of the ultra-short screening tools, the Whooley questions performed the best. Furthermore, the psychometric performance of the two Whooley questions is comparable to the 10-item EPDS, the K10 and the 9-item PHQ9. In addition to being ultra-short, it is a simple binary-scoring instrument that is possibly more feasible and acceptable for busy, low-resource primary care settings for use by non-specialist health workers than longer, Likert-type tools. Fig 2.

Ease of administration of the screening tool is likely to play a critical role in feasibility and implementability of screening [14]. Where health workers have limited time or numeracy skills, as may pertain to many LMICs, longer, Likert-type tools may be inaccurately used or avoided. Thus, although longer, Likert-type tools may perform better psychometrically than shorter, binary-type tools, these advantages may be outweighed by considerable logistical challenges.

In our study, the cut off scores of various instruments are also lower than those specified in other studies and in high-income countries. Research on the use of commonly used mental health screening instruments has found that it is not unusual for cut off scores to be lower in resource-constrained settings [16]. There is also variability in the sensitivity of cut-off scores depending on contextual factors which have to be taken into account, and researchers caution against the use of a single cut-off score [58]. Low cut-off scores could also yield higher numbers of false positives, which may be disadvantageous in settings where resources for mental health care are scarce. In addition to potentially creating undue burden on referral sources, programme design should take in to consideration the possible harm of creating unnecessary concern in women with false positive screens. This needs to be balanced with the clinical problem of false negative screening in which treatable psychopathology may go undetected. Caution must therefore be taken when determining whether greater sensitivity or specificity are desired, and researchers are encouraged to present a range of potential cut-off scores for validation research findings, which may be clinically useful [58].

### Strengths and limitations

This is one of the few studies to examine multiple screening tools simultaneously against a clinical diagnostic interview among pregnant women. It is also the first study to assess the performance of these tools in detecting both antenatal MDE and anxiety disorders, prompted by their high prevalence and comorbidity. However there are several limitations that warrant attention. First, women were only screened once, which limits the comparison of the screening instruments’ performance at various points across the perinatal period. Second, we only included one anxiety-symptom screening instrument and did not include commonly used anxiety symptom screening instruments such as the HADS, which may have yielded a better performance in detecting anxiety disorders. Third, the battery of screening instruments were administered in the same order and this may have primed responses to subsequent questions that were similar, though from different screening instruments. Following from this point, no analysis was conducted to examine differences in women’s scores based on order of presentation of the questionnaires, nor was inter-rater reliability testing conducted to ascertain the percentage of score variation between administrators of the screening instruments. Both of these factors could have affected study outcomes. Fourth, only one screening instrument, the EPDS, was developed to measure symptoms of depression specifically during the perinatal period. Lastly, while this study provides insights into initial screening for antenatal depression and anxiety, it does not address referral pathways and interventions–both of which are challenges in LMIC settings.

### Implications and recommendations

The EPDS, K10 and PHQ9 are valid screening instruments to detect antenatal anxiety and depression in low resource settings; however, their feasibility and practicality remains to be examined in non-research settings. Short and ultrashort instruments such as the K6 and the Whooley questions may also be valid when used for initial screening. The choice of optimal cut off scores that yield the best balance between sensitivity and PPV may need to be tailored to individual settings with higher cut-offs being recommended in resource-limited, primary-care settings.

Given that the ultra brief scales such as the Whooley questions perform similarly to longer scales such as the EPDS, one implication of this study is that ultra-brief scales may be used instead of longer ones–as a first line of screening. Where such ultra short screening instruments are used, it is advised to follow-up with more in-depth screening or clinical assessment. This is especially important considering the high rate of women expressing suicidal ideation and behaviour [59].

There may be concerns about implementing screening for CPMD in resource-constrained settings in LMICs. Although research shows that women are able to identify with or recognize symptoms when described, there are other factors such as low levels of literacy and low familiarity with test-taking which may impact self-report screening measures [16,60]. This needs to be taken into account when considering the development of screening protocols for such settings.

Where possible, screening needs to be integrated into antenatal care at primary care level and protocols must be put in place for referral for treatment and for follow up care. Effective screening is not only an activity for case identification, but has the potential to catalyze a process of further assessment and referral to treatment and care. Psychosocial assessment, which includes a broader enquiry into mental health and social circumstances, may be added to clinical symptom screening to inform better decisions about overall care [61,62].

More research is needed to validate screening tools or test their feasibility and acceptability and practical application in low resource settings. There also needs to be further research into the capacity of these tools to detect antenatal anxiety disorders in addition to MDE. There may be scope for the development of new screening tools for use in these contexts where they would need to be relatively short, easy to administer and to score.

## Conclusions

Universal screening for case identification of CPMD in low-resource environments may be conducted with a number of commonly used screening instruments. For detection of MDE and/or anxiety disorders, the K6 screening instrument may be the most sensitive, feasible and acceptable tool. The Whooley questions perform almost as well, and have the added benefit of being an ultrashort screen with a binary scoring system, which may improve feasibility and acceptability, where there are high patient volumes and low literacy levels. These questions may provide a relatively accurate, first-level screen within an integrated, stepped-care model of mental health service provision.

## Supporting information

S1 Table Sensitivity analysis of ROC output and cut-points against CPMD diagnosis. (DOCX)
