# Supplementary material for: Comparison of mental health screening tools for detecting antenatal depression and anxiety disorders in South African women
Source: PLoS One. 2018 Apr 18;13(4):e0193697. doi: 10.1371/journal.pone.0193697 (PMC5906008; doi:10.1371/journal.pone.0193697)
Supplement: S1 Table — (DOCX) [file pone.0193697.s001.docx]

**Supporting information**

**S1 Table. Sensitivity analysis of ROC output and cut-points against CPMD diagnosis.**

| **Screening tool** | **Cutpoint** | **Sensitivity** | **Specificity** | **Correctly classified** |
| --- | --- | --- | --- | --- |
| EPDS | >=10 | 87% | 58% | 66% |
|  | >=12 | 78% | 74% | 76% |
|  | >=13 | 75% | 78% | 77% |
|  | >=14 | 67% | 83% | 78% |
|  | >=15 | 59% | 89% | 79% |
| 3-item EPDS | >=2 | 84% | 57% | 66% |
|  | >=3 | 70% | 77% | 75% |
|  | >=4 | 51% | 89% | 77% |
| K10 | >=9 | 84% | 68% | 73% |
|  | >=10 | 82% | 72% | 75% |
|  | >=11 | 80% | 79% | 80% |
|  | >=12 | 75% | 82% | 80% |
|  | >=13 | 72% | 85% | 81% |
|  | >=14 | 70% | 87% | 82% |
| K6 | >=6 | 80% | 72% | 74% |
|  | >=7 | 75% | 80% | 79% |
|  | >=8 | 74% | 85% | 81% |
|  | >=9 | 69% | 88% | 82% |
|  | >=10 | 63% | 91% | 82% |
| PHQ9 | >=7 | 82% | 67% | 72% |
|  | >=8 | 73% | 72% | 73% |
|  | >=9 | 69% | 80% | 76% |
|  | >=10 | 66% | 86% | 80% |
|  | >=11 | 61% | 86% | 81% |
|  | >=12 | 53% | 92% | 80% |
|  | >=13 | 48% | 94% | 79% |
| PHQ2 | >=1 | 90% | 46% | 60% |
|  | >=2 | 75% | 69% | 71% |
|  | >=3 | 54% | 86% | 76% |
| Whooley questions | >=1 | 81% | 63% | 69% |
|  | >=2 | 66% | 87% | 80% |
| Whooley + help question | >=1 | 81% | 63% | 69% |
|  | >=2 | 73% | 82% | 79% |
|  | >=3 | 53% | 94% | 81% |
